# What is the Role of Caffeine in the Management of Preterm Infants?

**DOI:** 10.1007/s40746-025-00329-5

**Published:** 2025-07-02

**Authors:** David M. Rub, Eric C. Eichenwald

**Affiliations:** 1 Division of Neonatology, Children’s Hospital of Philadelphia, Philadelphia, PA USA; 2 Department of Pediatrics, University of Pennsylvania Perelman School of Medicine, 3401 Civic Center Blvd, Philadelphia, PA 19104 USA

**Keywords:** Caffeine, Apnea, Prematurity, Neonate

## Abstract

**Purpose of Review:**

To describe how clinical practice around caffeine therapy for apnea of prematurity has shifted in response to emerging data, with particular emphasis on changes in initiation timing, dosing strategies, and treatment duration.

**Recent Findings:**

Several new studies have begun to explore alternative approaches to caffeine therapy, including trials of caffeine initiation in the delivery room, increased loading and maintenance dosing, and extended use through later postmenstrual ages. Notably, the MOCHA and ICAF trials offer new insights into the potential risks and benefits of prolonging therapy beyond traditional discontinuation thresholds. These studies reflect growing interest in tailoring caffeine treatment to the evolving physiology of preterm infants, though long-term outcomes remain under investigation.

**Summary:**

Clinical use of caffeine has evolved far beyond the original CAP protocol, driven by physiologic rationale and early trial signals, but often outpaces the strength of the evidence. Future multicenter, randomized trials are essential to confirm safety and efficacy of earlier initiation, higher dosing, and extended duration and to ensure that practice refinements translate into durable improvements in preterm infant outcomes worldwide.

## Introduction

Since the 1970s methylxanthines have been the primary pharmacologic treatment for preterm infants with apnea of prematurity (AOP) [1, 2]. Caffeine, a derivative of theophylline, is the methylxanthine of choice for AOP treatment due its long half-life, oral bioavailability, and most importantly, its well studied safety profile [3]. Caffeine inhibits central adenosine receptors, increasing central respiratory drive, increases sensitivity to CO_2,_ and improves diaphragmatic contractility, decreasing the severity and frequency of apnea spells in preterm infants [3]. While the use of caffeine therapy in premature infants increased starting in the 1980’s, it was not until the Caffeine for Apnea of Prematurity (CAP) Trial that caffeine’s safety was rigorously evaluated [4].

The CAP trial was an international, multi-center, pragmatic double-blind, randomized control trial which evaluated the safety of caffeine by assessment of long-term clinical outcomes in preterm infants. In this trial, 2,006 infants with a birthweight of 500–1250 g were randomized within the first 10 days of life at their clinician’s discretion to receive either caffeine citrate (20 mg/kg loading dose, 5 – 10 mg/kg maintenance) or placebo until they reached a gestational age when apnea was no longer a concern. The primary outcome of the CAP trial was a composite of death, cerebral palsy, cognitive delay, deafness, or blindness at a corrected age of 18 to 21 months [4]. In addition, short-term secondary outcomes included major neonatal morbidities, such as development of bronchopulmonary dysplasia (BPD) or necrotizing enterocolitis (NEC). Notably, the CAP trial found that caffeine reduced BPD, facilitated earlier extubation, and improved survival without neurodevelopmental disability at 18–21 months. Caffeine was also associated with a transient reduction in weight gain presumably due to increased metabolic rate. Key findings from the original CAP trial and follow-up studies through school age are listed in Table 1. Since the CAP Trial, caffeine use in preterm infants for the prevention and treatment of apnea has become almost universal in high and moderate income countries.

**Table 1:** Follow-up and post-hoc studies from the CAP trial (Caffeine for Apnea of Prematurity) and their key outcomes

Study (Authors, Year)	Focus	Key Findings
CAP Trial Primary Outcome (Schmidt et al., 2006)	Caffeine vs placebo in 2006 infants < 1250 g; outcomes at 36 wks PMA	Caffeine reduced BPD (36% vs 47% oxygen at 36 wks) and facilitated earlier extubation (median 1 week earlier off support). No increase in mortality or major neonatal complications
CAP Trial 18–21 mo Follow-up (Schmidt et al., 2007)	Neurodevelopment at 18–21 months	Caffeine group had higher survival without neurodevelopmental disability (59.8% vs 53.8%). Lower rates of cerebral palsy (4.4% vs 7.3%) and cognitive delay. No difference in hearing or vision impairment
Age 5 Years Follow-up (Schmidt et al., 2012)	Outcomes at 5 years of age	No significant difference in combined outcome of death or disability (21.1% vs 24.8%, *p* = 0.09). Cognitive scores and behavior similar between groups. No late-emerging adverse effects of caffeine
Age 11 Years Follow-up (Schmidt et al., 2017)	Academic performance, motor, behavior at 11 years	No difference in academic failure or behavior problems. **Motor impairment was lower** in caffeine group (19.7% vs 27.5%, *p* = 0.009), suggesting lasting motor benefit. Neonatal caffeine deemed safe with no long-term harm

In the ensuing 40 years, multiple studies have been conducted to search for ways to optimize caffeine therapy. These include studying the timing of initiation of therapy, higher dosing than used in the CAP Trial, and extended time to discontinuation [5]. These studies, often observational or small randomized control trials, have influenced international clinical guidelines for caffeine use, which now include significant departures from the original CAP protocol [6–8].

In this review, we examine the emerging evidence guiding the departures form the CAP protocol within 3 main domains: earlier initiation, higher dosing strategies, and extended use. By synthesizing these evolving practices and the data supporting them, we aim to clarify the rationale behind current approaches and identify remaining gaps in evidence that ongoing trials seek to address.

## Optimal timing of caffeine initiation

One of the most significant practice changes in neonatology since the CAP trial has been the trend toward earlier initiation of caffeine in extremely preterm infants. In the CAP trial, infants could be enrolled up to 10 days of age, and the median age at caffeine start was around 3 days. The indications for initiation included treatment or prevention of apnea in non-intubated infants, or to facilitate extubation [4].

A post-hoc subgroup analysis of the CAP trial found that starting caffeine within the first 3 days after birth resulted in larger reductions in days of respiratory support and a 52% decrease in the rate of BPD compared to 23% in the group that received caffeine after 3 days [9]. Despite the concern that these results may reflect confounding by indication, as sicker infants are likely to trial extubation later, these results moved many centers toward administering caffeine prophylactically within the first 1–2 days of life for mechanically ventilated infants at high risk of apnea (gestational age < 28 weeks).

Following this shift in practice, several subsequent large observational studies further supported the practice of early caffeine initiation [10–12]. A recent systematic review and meta-analysis of both large, observational studies and several, small, single-center randomized trials found that early caffeine use (within 72 h of birth) was associated with decreased odds of retinopathy of prematurity (ROP), intraventricular hemorrhage (IVH) and BPD [13]. However, early caffeine use was also associated with increased mortality. This unexpected finding is most likely reflects survivor bias, but it underscores the critical need for rigorous, prospective studies to clarify the safety and efficacy of early caffeine administration in mechanically ventilated preterm infants. Of note, one such randomized, double-blind, placebo-controlled trial of early caffeine administration to preterm infants requiring prolonged mechanical ventilation was stopped early due to a trend towards increased mortality in the caffeine group [14].

Building on this trend toward earlier administration, additional studies have explored even more immediate caffeine delivery—beginning in the delivery room itself [15–17]. These small trials, focused on short-term physiologic outcomes, suggest potential benefits such as improved tidal volumes and systemic blood flow. However, evidence for this practice remains limited, and key questions remain about its safety and long-term clinical impact.

## Evolving caffeine dosing practices

The CAP trial firmly established standard dosing of caffeine (20 mg/kg load, 5–10 mg/kg/day maintenance) to be safe in treating apnea and improving outcomes in very low birth weight infants [4]. Since then, several small randomized trials have explored alternative dosing strategies, including higher loading doses (up to 80 mg/kg) and increased maintenance doses (up to 40 mg/kg/day), to evaluate short-term outcomes such as apnea frequency and extubation failure. Several of these studies, did show that higher caffeine dosing was generally associated with improvements in these short-term respiratory outcomes [18–20].

Multiple systematic reviews and meta-analyses conducted on higher caffeine doses—defined broadly as any dose exceeding CAP trial standards—were associated with reductions in BPD, without clear effects on mortality or long-term neurodevelopmental outcomes [21, 22]. However, because these meta-analyses grouped together varied definitions of “high” dosing, they do not clarify whether benefits are driven by higher bolus doses, increased maintenance, or both. Of concern, in one trial which used the highest loading dose (80 mg/kg), there was an increased risk of cerebellar hemorrhage and a lower seizure threshold in the high-dose group [23]. Larger, multi-center RCTs focused on safety and long-term effectiveness of different dosing regimens are currently in development to try and fill this evidence gap [24].

Another emerging dosing strategy is variable adjustment over time, independent of symptom burden. This strategy is based on caffeine pharmacokinetics in the preterm neonate. Due to immature hepatic enzyme activity, preterm infants metabolize caffeine at a much slower rate compared to term infants and adults [25]. In the first weeks of life, only a small fraction of caffeine is metabolized by the liver; most is excreted unchanged by the kidney. By ~ 34–36 weeks PMA, hepatic maturation accelerates caffeine clearance, shortening its half-life. This developmental change has clinical implications: a fixed maintenance dose of caffeine will yield progressively lower serum levels as a preterm infant matures and clears the drug faster [25]. The variable adjustment strategy empirically increases caffeine dose each week by 1 mg/kg to target stable caffeine troughs [26].

Despite evidence of short-term benefit of higher dosing strategies, current evidence remains insufficient to support routine use of these approaches outside of a research setting. There is a lack of robust safety data regarding long-term outcomes associated with higher loading or maintenance caffeine doses. Nevertheless, some international clinical guidelines have endorsed higher dosing regimens (e.g., Queensland Health, Australia: 20-80 mg/kg loading, 5–20 mg/kg/day maintenance), highlighting a disconnect between evolving practice patterns and the available safety data [6, 8]. Until adequately powered, multi-center trials establish the long-term safety of alternative regimens, the standard CAP protocol remains the only regimen with proven long-term safety.

## Duration of therapy and discontinuation

Determining the appropriate duration of caffeine therapy is another nuanced and evolving aspect of AOP management. In the CAP trial, the median postmenstrual age at caffeine discontinuation was 34 weeks. No studies have examined the optimal strategy for discontinuation of caffeine therapy. As such, most recommendations are based on expert opinion. This has led to wide variability in practice: a 2019 study of 304 NICUs (over 80,000 infants) found the mean PMA at caffeine discontinuation ranged from 32 to 37 weeks depending on the center [27]. In some units virtually all infants were off caffeine by 34 weeks, while in others many infants stayed on caffeine until 36–37 weeks or were discharged with home caffeine [27].

The American Academy of Pediatrics Committee on Fetus and Newborn clinical report on apnea of prematurity recommended that caffeine be stopped at 33 to 34 weeks postmenstrual age, or when apnea is no longer evident off positive pressure ventilation, whichever comes first [3]. These recommendations are based on the desire not to delay discharge to home, since most NICUs require a 5—7 day apnea free period off caffeine prior to discharge. Given the long half-life of caffeine, its discontinuation well before a baby is approaching discharge has been common practice [4]. However, it is well documented that some preterm infants may continue to experience intermittent hypoxia beyond 34 weeks PMA which is often clinically inapparent, which puts them at continued risk for hypoxic events at home [28].

In response, some have argued for extending caffeine treatment until closer to term age. Others have explored sending infants home on caffeine therapy, circumventing the need to stay in hospital solely for apnea monitoring.

Two recent multi-center RCTs have investigated the question of delayed caffeine discontinuation. The Moderately Preterm Infants with Caffeine at Home for Apnea (MOCHA) Trial enrolled infants 29 to < 34 weeks gestation who had been started on caffeine for AOP and reached 33–35 weeks PMA with a clinician driven plan to discontinue caffeine [29]. Infants were then randomized to continue caffeine therapy at 10 mg/kg/day or placebo through the remaining hospitalization and for 28 days following discharge home. The primary outcome was number of days of hospitalization after randomization. The study enrolled 827 preterm infants and found no difference in hospital length of stay (primary outcome). Infants receiving caffeine did achieve the 5-day apnea free interval quicker than the placebo group, but this did not affect time to discharge, as feeding difficulties were the rate-limiting step to discharge in this cohort.

In a much smaller randomized trial, the Intermittent Hypoxia and Caffeine in Infants Born Preterm (I-CAF) study enrolled infants < 30 weeks gestation at birth and randomized them at 32–36 weeks PMA to continue caffeine therapy until 43 weeks PMA, with a per-protocol doubling of the caffeine dose to 20 mg/kg/day at 36 weeks PMA [30]. The doubling of caffeine dose was driven by the PMA dependent changes in caffeine metabolism and clearance previously described above. Infants in this study were also on continuous pulse oximeter recording through the study period to measure exposure time to intermittent hypoxia.

In contrast to MOCHA, the ICAF trial found notable benefits to extended caffeine. Among 160 randomized infants, those who continued caffeine had less need for respiratory support and oxygen near term and went home sooner**.** By 36 weeks, only 4.9% of the caffeine group still required supplemental oxygen vs 17.9% of the placebo group (*p* = 0.009). Importantly, discharge was accelerated**:** mean PMA at discharge was 37.7 weeks for caffeine infants vs 38.5 weeks for placebo (*p* = 0.04), and the median time from randomization to discharge was 10 days shorter with caffeine (17 vs 27 days, *p* = 0.001). Extended caffeine also dramatically reduced intermittent hypoxic episodes, an effect that persisted until 42–43 weeks PMA [30].

The stark contrast in outcomes between MOCHA and ICAF are possibly explained by immaturity of infants enrolled in the ICAF study. Infants born at earlier gestational ages are more likely to suffer from extended apnea and intermittent hypoxia, and therefore more likely to benefit from extended caffeine therapy [28]. Despite these two studies, it the optimal time for caffeine discontinuation remains uncertain, and requires further investigation.

## Alternative mechanisms of action

**Fig. 1:**
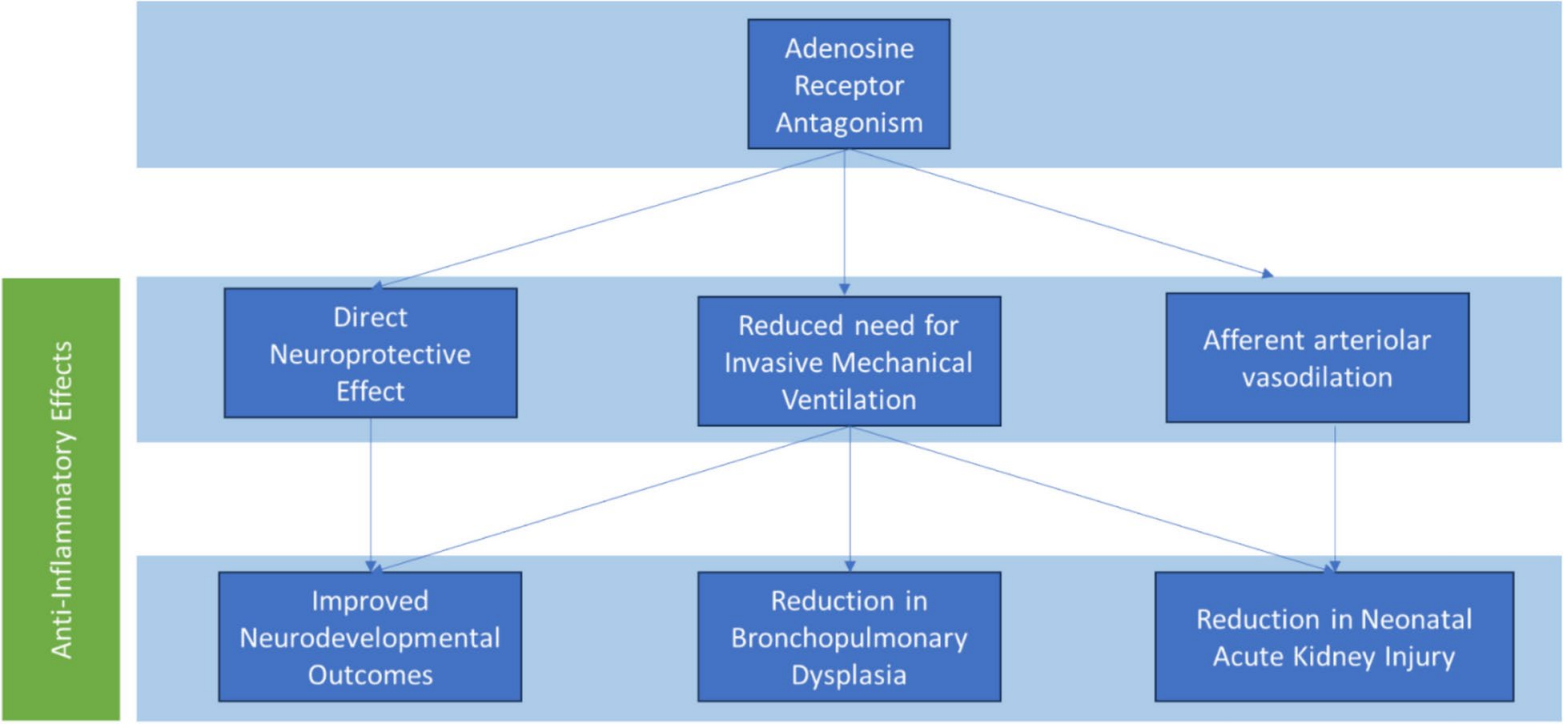
Mechanisms through which caffeine exerts its therapeutic effects in preterm infants

Post-hoc analyses from the CAP trial’s 18–21 month, 5-year and 11-year follow-up studies suggested most of the neurodevelopmental benefit of caffeine were attributable to its effects on the respiratory system—primarily through earlier discontinuation of invasive and non-invasive ventilation [31, 32]. However, this leaves a substantial portion of caffeine’s observed benefit unexplained, suggesting the presence of other physiologic or pharmacologic mechanisms. Beyond its known action on central respiratory control via adenosine receptor antagonism, caffeine also exhibits anti-inflammatory properties and exerts direct effects on multiple organ systems, including the brain and kidneys (Fig. 1).

Caffeine’s immunomodulatory effects are thought to be mediated, in part, through adenosine receptor antagonism [33] In a mouse model, caffeine attenuated the inflammatory response following hyperoxia exposure by reducing levels of pro-inflammatory cytokines and chemokines implicated in the pathogenesis of BPD [34]. A small, prospective observational study in preterm infants similarly found caffeine to be associated with decreased levels of pro-inflammatory cytokine [35]. Notably, this relationship appeared to be U-shaped: while moderate caffeine levels (10–20 µg/mL) were associated with reduced inflammation, serum concentrations exceeding 20 µg/mL were linked to a pro-inflammatory cytokine profile, including elevated TNF-alpha and IL-6 [35]. These findings raise important questions about the potential pro-inflammatory consequences of higher caffeine dosing.

Expanding on its potential neurological impact, studies are increasingly focusing on the role of caffeine in early brain development [36]. Investigations using amplitude-integrated EEG and neuroimaging techniques have demonstrated that caffeine may increase cortical activity, a marker of arousal, and improve white matter microarchitecture [37]. Ongoing follow-up studies of the CAP cohort into adolescence aim to determine whether neonatal caffeine exposure is associated with long-term academic or health outcomes, offering insight into possible enduring neurodevelopmental effects.

Parallel lines of research have suggested that caffeine-treated infants may experience lower rates of acute kidney injury by improving renal regional oxygen saturation^8^. Secondary analysis of the Assessment of Worldwide Acute Kidney Injury Epidemiology in Neonates (AWAKEN) study, demonstrated that administration of caffeine in the first 7 days of life significantly reduced the incidence of AKI in preterm neonates. Specifically, AKI occurred in 11.2% of neonates who received caffeine compared to 31.6% of those who did not, with an adjusted odds ratio of 0.20 (95% CI, 0.11–0.34) [38]. These findings suggest that caffeine has a protective effect against renal injury in preterm neonates, potentially due to its effects on renal hemodynamics and oxygenation. However, similar to the observational studies described earlier, these results should be interpreted with caution due to high risk of confounding by indication. Further research is needed to assess caffeine as a nephro-protective agent and optimize the timing and dosage of caffeine administration to maximize these effects.

## Global accessibility of caffeine therapy

Despite the well-documented benefits of caffeine therapy for preterm infants, its availability varies significantly across different healthcare settings worldwide. In high-income countries, caffeine citrate is a standard medication in neonatal intensive care units, but access remains limited in many low- and middle-income countries (LMICs) where the burden of preterm birth is highest [39].

Several barriers contribute to this disparity. First, although caffeine is a cost-effective strategy in high-income countries [40],the cost of pharmaceutical-grade caffeine citrate can be prohibitive in resource-limited settings [41]. While caffeine is inherently inexpensive, the specialized formulation for neonatal use carries premium pricing that may be unaffordable for many healthcare systems [40]. Furthermore, stable supply chains for specialized neonatal medications are often lacking in remote or underserved regions [42] and the infrastructure required for caffeine administration—including staff trained in neonatal care, monitoring equipment, and laboratory facilities for therapeutic drug monitoring when needed—may be insufficient [43].

Addressing these disparities requires multi-faceted interventions: development of affordable formulations, inclusion of caffeine in national essential medicine lists, strengthening of neonatal care infrastructure, and implementation of context-appropriate protocols for caffeine administration. International collaborations between academic centers, industry partners, and global health organizations could accelerate progress toward equitable access to this evidence-based therapy for all preterm infants, regardless of geographic location [44].

## Conclusion

The evolution of caffeine therapy since the landmark CAP trial represents a nuanced journey of clinical practice refinement guided by emerging evidence. While the CAP trial provided robust evidence for the safety of standard caffeine dosing at moderate postnatal ages, subsequent investigations have explored deviations from this protocol—earlier initiation, higher dosing, and extended duration—to improve outcomes for specific subpopulations of preterm infants.

While these evolving practices are physiologically and clinically compelling, they often outpace the strength of the evidence base. Ongoing and future randomized trials will be critical to validating these approaches and ensuring that expanding use of caffeine remains grounded in safety and efficacy. These future studies must also include infants historically excluded from the evidence base, including those born at less than 24 weeks gestational age or receiving chronic ventilation. The ongoing evolution of caffeine therapy illustrates how rigorous research, clinical observation, and thoughtful implementation can transform a simple intervention into a cornerstone of neonatal care that continues to improve outcomes for premature infants worldwide.

## Data Availability

No datasets were generated or analysed during the current study.
